# Assessment of knowledge, attitude and practice towards disposal of unused and expired pharmaceuticals among community in Harar city, Eastern Ethiopia

**DOI:** 10.1186/s40545-018-0155-9

**Published:** 2018-11-15

**Authors:** Yohanes Ayele, Mulu Mamu

**Affiliations:** Department of Clinical Pharmacy, School of Pharmacy, College of Health and Medical Sciences, Haramaya University, P.O. Box 235, Harar, Ethiopia

**Keywords:** Pharmaceuticals, Disposal practices, Unused, Expired, Knowledge, Attitude

## Abstract

**Background:**

Pharmaceuticals are produced and consumed in increasing volume every year. Unfortunately, not all medications that go in to the hand of the consumers get consumed; large quantities remain unused or expire. The accumulation of medication at household and unsafe disposal of unwanted medicines could lead to inappropriate medicine sharing, accidental childhood poisonings and diversion of medicines to illicit use.

**Methods:**

A descriptive cross-sectional study was conducted among 695 residents in kebele(ward) 16 of Jinela woreda(district), Harar city from February 27–April 27. A multi-stage sampling technique was used to select individual households. Face-to-face interview using structured questionnaires were conducted to collect data from each study subject. The cleaned data was entered in to epidata analyzed using SPSS version 20 software. Descriptive statistics on sample characteristics was computed including frequencies and percentage and presented using tables and figures.

**Results:**

Most participants displayed correct understanding toward medication waste (72.9%) and its effect on environmental if disposed improperly (86%). A large portion of the respondents did not know about drug-take-back system 464 (66.9%). In order to minimize the entry of pharmaceuticals into environment, 68.6% of the participants suggested the need for proper guidance to the consumer. Majority of the respondents believed risk related to the presence of unwanted drug in home, potential harm to children, lack of adequate information on safe disposal practice and need for take-back program. Approximately 66% of the respondents had unused medicine stored at home and the common types of medicines kept in households were analgesics (62.7%) and antibiotics (24%). Preferred ways of disposal of both unused and expired medicine was throwing away in household garbage (53.2%) and two third of them disposed the pharmaceuticals in its original package and dosage form.

**Conclusion:**

In present study, there was high practice of keeping medication at home and most disposal approach indicated by the participants was not recommended methods. Awareness about proper disposal of unused and expired medicines among the public should be created. Guidelines on safe disposal are required and an organized method of collecting unused and expired pharmaceuticals needs to be introduced.

## Background

A large volume of pharmaceuticals and health care products are used annually for diagnosis, treatment or prevention of health conditions. However, not all products that go in to the hand of consumers get consumed; large quantities go unused or expire. Medications may accumulate in households for a variety of reasons: improvement of the patient’s medical condition, oversized medication packages, death of the patient and change in prescription due to side effects or lack of therapeutic effect, poor adherence as the result of patients doubting the need for medication, fear of adverse effects or forgetfulness also contribute to the medication wastage [1, 2] Improper disposal of medications pose a significant environmental risk such as on the water system. A long term environmental exposure to pharmaceuticals could lead to hazardous effect especially on vulnerable populations, including pregnant women, newborn, and children [3]. In addition, evidence shows that the presence of antibiotics in environment may lead to antibiotic resistance [4]. Furthermore, storage of unwanted or unused medication in the household provides an opportunity for misuse and abuse when one inadvertently takes them [5]. Subsequently, the disposal practice of unused medicines has become a worldwide challenge catching the attention of policy makers, health professionals, pharmaceuticals companies and the community in general.

Study conducted in different areas indicated not only diverse practice of disposal of unused medicine but also respondents were not fully aware of appropriate approaches. For example, study conducted in Kenya [6] and Nigeria [7] revealed that the most preferred disposal method for unused pharmaceuticals was throwing in garbage bins followed by flushing in the toilets. In other study, the respondents kept the drug in their home because they were not sure what to do with them and some other shared to friends and families [8, 9]. These studies clearly show that most of the respondents lack of awareness in proper methods of dispose of unwanted medicines.

Globally, safe disposal of expired, unwanted, or unused medications particular by the consumers is of high concern. Many developed countries have programs aimed at disposal of unused medicines. For instance, in Australia and Canada’s there has been the National Return and Disposal of Unwanted Medicines Project which is fully supported by the government and pharmaceutical industry [10]. The drug take-back programs are also common in the United Kingdom and Sweden [11].

Unfortunately in African countries, programs or system advocating safe disposal practices of unused medicines are still limited. In Ethiopia, there are no national policies that are aimed to control safe disposal of unused medicines and create public awareness on the issue. To encourage safe and appropriate disposal of pharmaceutical by community as well as bring the issue to the concern of the government, understanding the level of knowledge and attitude of community toward disposal of unused pharmaceuticals would be important step. Moreover, information on commonly employed method of disposal of unused pharmaceuticals would help the process of awareness creation on proper ways of removal of unused medicines. Therefore, this study was conducted to assess the knowledge, attitude and practices toward disposal of unused and expired pharmaceuticals among households in Harar city, Eastern Ethiopia.

## Methods

### Study design and description of study settings

The descriptive cross-sectional study design was conducted in Harar city, Eastern Ethiopia from February 27–April 27, 2018. Harar is located 526 km from Addis Ababa, the capital of Ethiopia. The region, Harar, is structured with 9 woredas(districts) that comprises 36 kebeles(wards). Harar city is composed of 6 woredas and 19 kebeles.

### Study design

The study was conducted through face-to-face interview using structured questionnaires to assess the knowledge, attitude andpractices towards disposal of unused and expired pharmaceuticals among community in Harar city, Eastern Ethiopia.

### Population

The source population was all households of Harar City and one resident from each house in selected study kebeland available during study period was included in this study. Residents who are less than 18 years old and unable to give interview were excluded from the study.

### Sample size determination and sampling technique

**Fig. 1:**
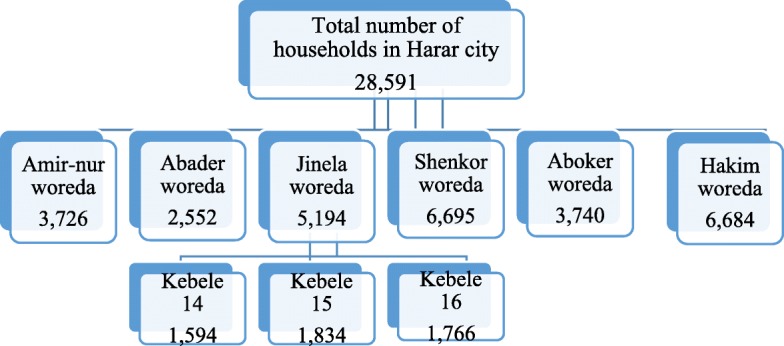
The schematic presentation of a multi-stage sampling technique used to select representative study kebel in Harar city, 2018

The sample size was determined using the single proportion formula and assuming *p* value of 50%, margin of error 5%, confidence interval of 95%. By considering design effect and non-respondents a total of 695 sample size was used in the study. A multi-stage sampling technique was used to select households. Jinela woredawas selected from all woredas of Harar city by using a simple random sampling technique. Kebele 16 was selected from all kebeles of Jinela woreda by using similar approach (Fig. 1). The study unit, households in kebele 16, was selected using a systematic sampling technique.

### Data collection procedure

Data was collected through interviews using structured questionnaires adapted from previous studies [8, 9] and modified to suit our purpose. Both close and open ended questionnaires were prepared in English language including all relevant variables based on the objectives of study. The tools used have four section designed to address; sociodemographic characteristics, knowledge, attitude and practice of participants toward unused and expired medications disposal. The final English version of questionnarieswere translated to Amharic and Oromiffa language by expert user of respective language.The translation was made by considering the conceptual equivalence of each questions and typical respondents. The questionnaires were translated back to English by independent translators to check consistence. Furthermore, pretest was conducted on the study population and important modifications were made accordingly.. Finally, the data was collected by two individuals, graduating pharmacy students.By training it was assured that the interview was effective and collector got the theme clearly..

### Data processing and analysis

The cleaned data was entered in to epidata and analyzed using SPSS version 20 software. Descriptive statistics on sample characteristics was computed including frequencies and percentage and presented using tables and figures.

## Results

### Socio-demographic characteristics of the participants

Almost all (694) approached individuals agreed to participate in the study giving 99.8% response rate. Among the total respondents, 368 (53.0%) were men and 326 (47.0%) were women. The majority were married 362 (52.2%) and most of the respondents had a monthly income of less than 6900 Ethiopian birr (250 USD) (Table 1).

**Table 1:** Socio-demographic characteristics of participants in Harar City, Eastern Ethiopia from February 27–April 27, 2018 (*n* = 694)

Variable	Frequency (%)
Age (years)	
18–24	193 (27.8)
25–35	214 (30.8)
35 and above	287 (41.4)
Gender	
Male	368 (53.0)
Female	326 (47.0)
Religion	
Orthodox	440 (63.4)
Muslim	146 (21.0)
Protestant	99 (14.3)
Others^b^	9 (1.3)
Educational	
Illiterate	50 (7.2)
Primary (1–8)	114 (16.4)
Secondary (9–12)	275 (39.6)
College and above	255 (36.7)
Marital status	
Single	272 (39.2)
Married	362 (52.2)
Divorced	35 (5.0)
Widowed	25 (3.6)
Occupations	
Self employed	362 (52.2)
Governmental employee	137 (19.7)
Student	91 (13.1)
Housewife	81 (11.7)
Others^c^	18 (2.6)
Monthly income (ETB)^a^	
< 1380	222 (32)
1381–6900	431 (62.1)
6901–13,800	41 (5.9)

^a^Classification is according to WHO income level scale for developing countries; ETB: Ethiopian birr; ^b^Catholic, Jehovah Witness and Traditional. ^c^daily labors, retired, no job

### Participants knowledge of unused and expired pharmaceuticals disposal

As presented in Table 2, the majority of the respondents 506 (72.9%) knew about medication waste. On the other hand, a large portion of the respondents did not know about drug-take-back system 464 (66.9%). A large share of the respondents (86%) correctly responded that improper disposal of unused and expired medicine could have detrimental effects on the environment. In order to minimize the entry of pharmaceuticals into environment, 68.6% of the participants suggested the need for proper guidance to the consumer. In response to a question about how to create the awareness among community, 49.57% answered that the best source is electronic media, 24.50% respondents mentioned Physicians. Surprisingly, only 8.5% of the participants held pharmacist responsible.

**Table 2:** Participants knowledge of unused and expired Pharmaceuticals disposal in Harar City, Eastern Ethiopia from February 27–April 27, 2018 (*n* = 694)

Questions/statements	*n* (%)
Do you know about medication waste?	
Yes	506 (72.9)
No	188 (27.1)
Do you ever read medicines disposal instructions?	
Yes	327 (47.1)
No	367 (52.9)
Do you know about “drug-take-back system”?	
Yes	230 (33.1)
No	464 (66.9)
Do you know that misused/repeated change or failing to complete antibiotics may cause drug resistance?	
Yes	542 (78.1)
No	152 (21.9)
Improper disposal of unused and expired medicines can affect the environment and health.	
Yes	597 (86.0)
No	97 (14.0)
How could be hazardous effect of unused and expired medicines minimized or controlled?	
Providing proper guidance to the consumer	476 (68.6)
Prescribing in quantities and for duration that ensure patient compliance	123 (17.7)
Lowering the number of prescribed medicine by doctor	32 (4.6)
Donating or sharing the unused medicines	34 (4.9)
Other^a^	29 (4.2)
Stakeholder for creating the awareness among community about proper disposal of unused and expired medicines	
Electronic Media	344 (49.6)
Physician	170 (24.5)
Pharmacy	59 (8.5)
Newspaper	35 (5.0)
All sources	78 (11.2)
Other^b^	8 (1.2)

^a^keeping in safe place, disposing in toilet, burning ^b^health bureau, environmental agency, drug regulatory agency

### Participants attitude toward unused and expired pharmaceuticals disposal

Just more than half of the respondents (52.4%) “Strongly agreed” about potential risks related to that presence of unused and expired medicines at home. Further, 61.7% of the respondents also “strongly agreed” that children are more at danger with unused and expired medicines while 38.8% of the respondents “strongly agreed” that lack of adequate information on safe disposal practices. With regards to take back programs, the respondents gave one sided opinions where 46.8% and 30.4% participant said programs should be mandatory; “strongly agreed” and “agreed” about the importance of the program respectively (Table 3).

**Table 3:** Perceptions on unused and expired Pharmaceuticals among Households in Harar City, Eastern Ethiopia from February 27–April 27, 2018 (*n* = 694)

Statement	Strongly disagree *n*(%)	Disagree *n*(%)	Neutral *n*(%)	Agree *n*(%)	Strongly agree *n*(%)
Unused and expired medicines present potential risks at home	33 (4.8)	30 (4.3)	7 (1.0)	260 (37.5)	364 (52.4)
Children are more vulnerable to the risks of associated with unused and expired household medicines	22 (3.2)	17 (2.4)	6 (0.9)	221 (31.8)	428 (61.7)
There is lack of adequate information on safe disposal of unused and expired household medicines	44 (6.3)	43 (6.2)	26 (3.7)	312 (45.0)	269 (38.8)
Doctors and healthcare professionals do provide advice on safe disposal of unused and expired household medicines	122 (17.6)	167 (24.1)	59 (8.5)	239 (34.4)	107 (15.4)
Take-back programs of unused and expired medicines should be mandatory	41 (5.9)	63 (9.1)	54 (7.8)	211 (30.4)	325 (46.8)

### Participants practice of unused and expired pharmaceuticals disposal

Approximately, two third of the participants had unused medicine at their home during the study period. The most preferred disposal practice for unused and expired pharmaceuticals was throwing in household garbage (53.2%). Interestingly, only 1% of the respondents perceived that returning unused medicines to the pharmacies would be the appropriate disposal practice. Around two third of respondents discarded the expired medications in its original package and dosage form, 15.4% did not know about the practice of expired medication disposal, 12.2% crushed before disposal (Table 4).

**Table 4:** Disposal practice of unused and expired pharmaceuticals among households in Harar City, Eastern Ethiopia from February 27–April 27, 2018 (*n* = 694)

Questions	*n* (%)
Did any quantity of purchased medicine remain unused at your home?	
Yes	459 (66.2)
No	235 (33.8)
What do you do with the unused medicines?	
Throw away in household garbage	369 (53.2)
Flush unused medications in toilet/sink	166 (23.9)
Keep at home until expired	111 (16.0)
Burn	15 (2.2)
Donate to hospital	13 (1.9)
Give to friends or relatives	13 (1.9)
Return back to pharmacy	7 (1.0)
What do you do with the expired medicines?	
Throw away in household garbage	369 (53.2)
Flush expired medications in toilet/sink	258 (37.2)
Return back to pharmacy	15 (2.2)
Give to friends or relatives	3 (0.4)
I don’t know what to do	27 (3.9)
Other^a^	22 (3.2)
Do you separate unused medicines before disposal?	
Yes	446 (64.3)
No	248 (35.7)
Way of discarding expired medicines	
Crashed before discarding	85 (12.2)
Diluted with water	41 (5.9)
As it is	461 (66.4)
I don’t know what to do	107 (15.4)

^a^land fill, burn, keep at home

### Respondents practice on unused and expired medication disposal

**Fig. 2:**
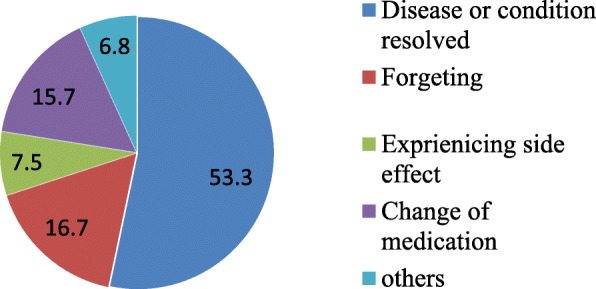
Respondents reason for purchased medicine remaining unused at home in Harar City, Eastern Ethiopia from February 27–April 27, 2018 (*n* = 459)

Reasons for possessing unused medication were mostly due to a resolved/improved disease or symptoms (53.31%) and forgetting to take (16.71%) (Fig. 2).``

**Fig. 3:**
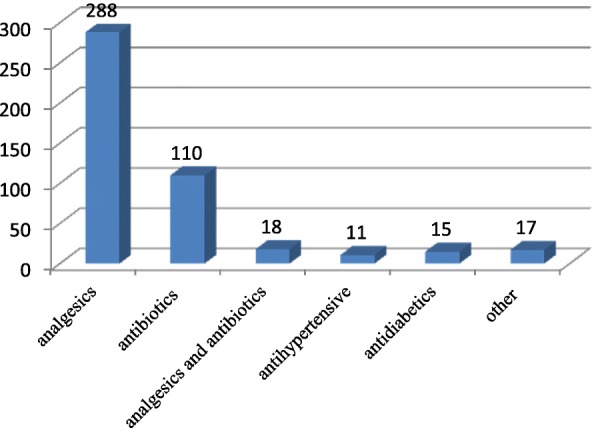
Type of pharmaceuticals remained unused at home among households in Harar City, Eastern Ethiopia from February 27–April 27, 2018 (*n* = 459)

The most common types of pharmaceuticals kept in households were analgesics (62.7%) followed by antibiotics (24%) (Fig. 3).

## Discussion

The aim of this study was to assess the knowledge, attitudes and disposal practices of unused and expired pharmaceuticals among households in Harar city. Most participants displayed correct understanding toward medication waste and its effect on environmental disposed improperly. However, most respondents were not aware of drug-take back system and had various opinions in ways of controlling or minimizing the effect of unused medicine. Majority of the respondents agreed in lack of adequate information on safe disposal practice. A large portion of participants kept drug at their home during the study period. Analgesics were the most common drugs found at home. Preferred ways of disposal of both unused and expired medicine was throwing away in household garbage as it is.

In this study, a large share of the respondents correctly understood detrimental effects of improper disposal of unused and expired medicine on the environment. This finding is higher than a study conducted in Karachi City [12] and Serbia [13]. However, it is significantly lower than study conducted in Kabul where almost entire sample felt that improper disposal of unused and expired medicines can affect the environment and health [9]. This might be due to lack of awareness creation programs and lack of effort from responsible governmental bodies to create awareness about the negative impacts of improper disposal of unused and expired medicines.

In this study, a large portion of the respondents did not know about drug-take-back-system. Although this result is much encouraging compared to study done elsewhere [14, 15], it shows lack of understanding about one the effective method of disposal of unused and expired medicine. This might be due to lack of an already established drug-take-back-system in Ethiopia, particularly in Harar city.

In present study, about two third of participants suggested need for proper guidance on disposal of unused and expired medicine while minor portion of the participants suggested prescribing less quantity. This result is inconsistent with a study conducted in Karachi City [12]. This finding indicates poor involvement of health care professional in creating awareness and guiding the consumers on proper disposal practice.

In our study, majority of the respondents agreed on lack of adequate information on safe disposal practice. Around half of participants denied receiving any information on safe disposal of unused and expired medicine from physicians and other health care professional. This finding is consistent with studies done elsewhere [16, 17]. This report implies need for public education to improve consumers’ awareness through various ways by responsible bodies including health care professionals, mass media, environmental agency.

In current study about two third of the respondents had leftover, unused or unwanted medications which is comparable with a study conducted in Gujarat [17] but slightly higher than study conducted among Serbian households (44.4%) [13]. This result, however, is significantly lower than study conducted in Kuwait [18] and Kabul [9]. This difference might be due to different systems and educational programs available in different countries. This increased storage of unused or expired medication in the household should be given emphasis as it can lead to irrational drug use since most people keep unused medications at home for future use or to share to friends/family members. It can also leads to accidental childhood poisonings.

In this study, the common types of medications kept in households were analgesics followed by antibiotics. This report is much higher than studies done elsewhere Nigerian [7], India [17] and USA [19] where the share of aforementioned category of the drugs had less share. This difference may be due to high prevalence of self-medication practice in Harar city. The increased presence of antimicrobials at home of should get serious consideration since it may add up to the problem of antimicrobial resistance.

In current study, the frequently stated reason for having leftover or unwanted medications was improvement in medical condition or resolved medical condition. This finding is similar to the finding from New Zealand [20] and Ghana [8]. However, it is different from study conducted in Kuwait where change or discontinuation of medication by the doctors’ was the main reason for having unused medicine at home [8, 18]. This report is worrying since the stated reason might be due to non-adherence.

In present study, the most preferred disposal practice for both unused and expired medicine was throwing in household garbage followed by flushing in toilet. This finding is consistent with study done elsewhere [6, 8, 20, 21]. In this study, very few people considered returning to pharmacy or health professionals as appropriate way of disposing unused or expired medicine. This might be due to lack of awareness about proper disposal of medications. This reasoning become more evident when we compare those findings with study conducted in Sweden where just under half of participants returned unwanted medication back to pharmacy [11]. The difference seen might be due to existence of system that encourage proper disposal of unused medicine in later country.

Around two third of respondents in the current study discarded or was willing to discard the expired pharmaceuticals in its original package and dosage form. Similar finding was reported elsewhere [8, 12]. This approach is contradicting with recommended ways of discarding expired medicine. For example, FDA [22] recommends crushing or dissolving in water and deletion of all personal information from medicine packaging. This approach is very important since it can prevent the drug re-utilization by scavengers.

### Limitation

This study should be interpreted cautiously for many reasons. Its generalizability is questionable since the study was conducted only in one center. In addition, since this is descriptive cross sectional design we were not able to identify associated factors with knowledge, attitude and practice of the participants.

## Conclusion

In present study, there was high practice of keeping unused medication at home.although majority of the them are aware of potential risk associated to the presence it. There was lack of adequate information on safe disposal practice and most respondent mentioned the need for system that encourage safe disposal of unwanted pharmaceuticals such as “drug take-back program”. In this study, most preferred methods for disposal of unused and expired medicines most were not recommended method.

## Abbreviations

FDA: Food and Drug administration
SPSS: Statistical Package for Social Sciences
USA: United State of America

## References

[1] Bound JP, Voulvoulis N (2005). Household disposal of pharmaceuticals as a pathway for aquatic contamination in the United Kingdom. Environ Health Perspect.

[2] Vogler S, Leopold C, Zuidberg C, Habl C (2014). Medicines discarded in household garbage: analysis of a pharmaceutical waste sample in Vienna. J Pharm Policy Prac.

[3] Daughton CG (2003). Cradle-to-cradle stewardship of drugs for minimizing their environmental disposition while promoting human health. II. Drug disposal, waste reduction, and future directions. Environ Health Perspect.

[4] Costanzo SD, Murby J, Bates J (2005). Ecosystem response to antibiotics entering the aquatic environment. Mar Pollut Bull.

[5] Beirens TM, van Beeck EF, Dekker R, Brug J, Raat H (2006). Unsafe storage of poisons in homes with toddlers. Accid Anal Prev.

[6] Angi’enda SA, Bukachi SA (2016). Household knowledge and perceptions on disposal practices of unused medicines in Kenya. J Anthropol Archaeol.

[7] Auta A, Omale S, Shalkur D, Abiodun AH (2011). Unused medicines in Nigerian households: types and disposal practices. J Pharmacol Pharmacother.

[8] Osei-Djarbeng SN, Larbi GO, Abdul-Rahman R, Osei-Asante S, Owusu-Antwi R (2015). Household acquisition of medicines and disposal of expired and unused medicines at two suburbs (Bohyen and Kaase) in Kumasi – Ghana. Pharma Innov J.

[9] Bashaar M, Thawani V, Hassali MA, Saleem F (2017). Disposal practices of unused and expired pharmaceuticals among general public in Kabul. BMC Public Health.

[10] Tong AY, Peake BM, Braund R (2011). Disposal practices for unused medications around the world. Environ Int.

[11] Persson M, Sabelstrom E, Gunnarsson B (2009). Handling of unused prescription drugs--knowledge, behaviour and attitude among Swedish people. Environ Int.

[12] Ahsaan Ahmed, Nousheen Mushtaq, Muhammad Tariq, Maliha Durrani SA, Muhammad Arif, Yasmeen G. Disposal practices of unused and expired pharmaceuticals in Karachi and their impact on health and environment. JUMDC 2013;4(2).

[13] Paut Kusturica M, Tomas A, Tomic Z, Bukumiric D, Corac A, Horvat O (2016). Analysis of expired medications in Serbian households. Slov J Public Health.

[14] Zubair Khalid labu, Mir Md Abdullah Al-Mamun, Md. Harun-or-Rashid, Sikder K. Knowledge, Awareness and disposal practice for unused medications among the students of the Private University of Bangldesh. J Biomed Pharm Res 2015;2(2):26–33.

[15] Azad AK, Ansary RH, Akhter A, Mostofa Al-Mamun SM, Uddin M, Rahman MM (2012). Disposal practice for unused medications among the students of the International Islamic University Malaysia. J Appl Pharm Sci.

[16] AlAzmi Aeshah, AlHamdan Hani, Abualezz Rayf, Bahadig Faiz, Abonofal Noha, Osman Mohamed (2017). Patients’ Knowledge and Attitude toward the Disposal of Medications. Journal of Pharmaceutics.

[17] Sonowal S, Desai C, Kapadia JD, Desai MK (2016). A survey of knowledge, attitude, and practice of consumers at a tertiary care hospital regarding the disposal of unused medicines. J Basic Clin Pharm.

[18] Abahussain EA, Ball DE, Matowe WC (2006). Practice and opinion towards disposal of unused medication in Kuwait. Med Princ Pract.

[19] Law AV, Sakharkar P, Zargarzadeh A, Tai BWB, Hess K, Hata M (2015). Taking stock of medication wastage: unused medications in US households. Res Soc Adm Pharm.

[20] Braund R, Peake BM, Shieffelbien L (2009). Disposal practices for unused medications in New Zealand. Environ Int.

[21] Al-Shareef F, El-Asrar SA, Al-Bakr L, Al-Amro M, Alqahtani F, Aleanizy F (2016). Investigating the disposal of expired and unused medication in Riyadh, Saudi Arabia: a cross-sectional study. Int J Clin Pharm.

[22] United States Food and Drug administration. How to dispose unused medications, 2011. Cited[15 July 2018]. Available from: www.fda.gov/forconsumers/consumerupdates/ucm101653

